# Characterization of Patients with Allergic Rhinitis to Ragweed Pollen in Two Distinct Regions of Romania

**DOI:** 10.3390/medicina55110712

**Published:** 2019-10-24

**Authors:** Ioana Corina Bocsan, Ioana Adriana Muntean, Corina Ureche, Raluca Maria Pop, Maria Adriana Neag, Octavia Sabin, Diana Deleanu, Anca Dana Buzoianu

**Affiliations:** 1 Department of Pharmacology, “Iuliu Hatieganu” University of Medicine and Pharmacy, Toxicology and Clinical Pharmacology, 400337 Cluj Napoca, Romania; bocsan.corina@umfcluj.ro (I.C.B.); raluca_parlog@yahoo.com (R.M.P.); meda_neag@yahoo.com (M.A.N.); octaviasabin@gmail.com (O.S.); abuzoianu@umfcluj.ro (A.D.B.); 2 Department of Allergology and Immunology, “Iuliu Hatieganu” University of Medicine and Pharmacy, 400146 Cluj Napoca, Romania; adrianamuntean77@gmail.com (I.A.M.); deleanudiana@yahoo.com (D.D.); 3 First Internal Medical Department, “George Emil Palade” University of Medicine, Pharmacy, Sciences and Technology, 540136 Târgu Mureș, Romania

**Keywords:** allergic rhinitis, ragweed, *Ambrosia artemisiifolia*, sensitization, asthma

## Abstract

*Background and objectives:* Ragweed pollen is a major source of allergen, which has rarely been observed in Romania until now. In this study, we evaluated the symptoms and associated factors in patients with allergic rhinitis to ragweed pollen in two distinct regions of Romania. *Materials and Methods:* We evaluated the records of patients newly diagnosed with allergic rhinitis induced by ragweed pollen in two allergological centers from North-West (NW) and Central parts of Romania between 2013 and 2015. The patients were clinically evaluated regarding disease length, presence, and severity of the allergic rhinitis symptoms and the association with other allergic manifestations (asthma and conjunctivitis). *Results:* The sensitization to ragweed was significantly higher in the NW part compared to the Central part (18.27% vs 4.1%, *p* < 0.001). More patients with monosensitization to ragweed pollen were observed in the NE center (27%) compared to the Central one (20.7%). Patients with monosensitization to ragweed pollen presented more severe forms of rhinitis (70% vs 31.5%, *p* = 0.02) in the NW part compared to polysensitized patients. The total symptoms score was significantly higher in patients from the Central part compared to the NW part (9.21 ± 2.01 vs 5.76 ±1.96, *p* < 0.001). Bronchial asthma was associated at a similar frequency to allergic rhinitis in both centers, but it was more frequently observed in monosensitized patients in the NW center. Allergic conjunctivitis was more frequently reported by patients from the Central part (75.86 vs 41.9, *p* = 0.02), while in the NW region it was noticed more commonly in monosensitized patients (65% vs 33.33, *p* = 0.02). *Conclusions:* Allergic rhinitis to ragweed pollen has been more frequently reported in the NW part of Romania. Patients with severe forms of rhinitis were observed in the central part, while in the NW the severe forms of disease were reported by patients with monosensitization. Ragweed pollen is intensely allergogenic and determines association of ocular and asthma symptoms. Co-sensitization increases the risk of asthma association.

## 1. Introduction

Respiratory allergies, like allergic rhinitis and bronchial asthma, are chronic diseases with high social impact worldwide, and epidemiological data has shown an increase in their incidence and prevalence [1,2]. Specific clinical manifestations could be extremely unpleasant, affecting the patients’ quality of life. In severe forms of allergic rhinitis, other non-nasal symptoms could be associated: ocular symptoms, asthma symptoms, headache, and sleep disturbances [3].

The prevalence of allergic rhinitis to pollen is estimated at 40% in the general population of Europe [1]. Total social costs of allergic rhinitis to pollen, quantified in the number of days of absenteeism from work or school, decreased work productivity, the number of visits to an allergologist and/or to an emergency department, and treatment costs are high enough to consider this disease as a real health problem in Europe and worldwide.

Weeds represent a major source of pollen, *Ambrosia artemisiifolia* being the most allergenic one. *Ambrosia artemisiifolia* (commonly known as ragweed) is an invasive annual herbaceous plant which originated from North America [4]. Ragweed was introduced In Europe in the 19th century, but become widely spread after 1900 due to an import of contaminated grains and seeds, from North America and its distribution is an on-going process [5]. The sensitization rate to ragweed is above 2.5% in general population in Europe [6]. But in some regions like Hungary, the Rhone Valley in France, and Northern Italy, the prevalence of ragweed sensitization varies considerably, reaching up to 60%–70% [6,7].

Ragweed is highly invasive and harmful for human beings because each plant produces a large amount of pollen (<1 billion grains/season) [8], which is highly allergenic; even low exposure may trigger a severe allergic reaction [9]. The peak of the season for ragweed pollen is in late summer and autumn in Romania [4]. Ragweed pollen grains can be transported over hundreds of kilometers by air, so they can induce allergy symptoms in areas where the ragweed plant is not widespread. Allergy to ragweed usually manifests clinically as allergic rhinitis or conjunctivitis, sometimes with associated asthma symptoms [10]. Despite this increasing health problem, there are few studies that have analyzed the impact of ragweed sensitization on the clinical manifestations of allergy, especially in Romania [4,11,12]. Most of the studies focused on determining the pollen level in the air and its correlation with the severity of manifestations. Sometimes it is difficult to measure the pollen amount in the air, so it is necessary to better understand the clinical picture of ragweed allergy.

The aim of the study is to characterize from the clinical point of view the patients with allergic rhinitis to ragweed and to identify possible associated factors that may predict the severity of manifestations, in two distinct centers from Romania.

## 2. Materials and Methods

### 2.1. Study Design, Site and Ethical Approval

The study was observational, analytic, and retrospective. The study was conducted in two Allergology Departments, one in Satu Mare, in the North-West (NW) region of Romania, near the borders to Ukraine and Hungary, and the second one in the Central part of the country, in Cluj Napoca. The study protocol was approved as 08.03.2017 by the Ethic Committee of “Iuliu Hatieganu” University of Medicine and Pharmacy (ID number 105/08.03.2017). The study was done in accordance with the Declaration of Helsinki.

### 2.2. Patient Evaluation

All the patients newly diagnosed with allergic rhinitis between 2013 and 2015 were included in the retrospective analysis. In the first center from Satu Mare, data from 405 patients with allergic rhinitis were analyzed, while in the second group from Cluj Napoca, 706 patients were included. Diagnoses of allergic rhinitis was done using international criteria [3], based on clinical evaluation and the skin prick test.

From patients files, the following data were registered: age of patients at the presentation moment, the length of allergic rhinitis, living area (urban or rural), the presence and severity of allergic rhinitis symptoms, and the associated allergic manifestations: bronchial asthma and allergic conjunctivitis. The diagnoses of asthma and allergic conjunctivitis were clinically established based on patient symptoms, according to international guidelines [3]. The authors recorded the presence of asthma and/or conjunctivitis symptoms during the ragweed pollen season, when the patients presented rhinitis manifestations.

The patients with allergic rhinitis to ragweed pollen were examined during the pollen season (August-September) when they had clinical symptoms of allergic rhinitis. The specific symptoms of allergic rhinitis-sneezing, rhinorrea, nasal congestion, nasal itching, and ocular itching were evaluated on a scale from 0 (lack of the symptom) to 3 (severe symptom). Based on symptoms scores, the total symptoms score (TSS) was calculated. A TSS < 6 represents a mild form of allergic rhinitis, while TSS ≥ 6 represents a moderate to severe form [3].

The allergological evaluation also included the skin prick test (SPT). The SPT was performed according to international guidelines [13] and the particularities of exposure to allergens in Romania. The tested panel included: house dust mites, mix grass pollen, cereals pollen, betulacee pollen, weed pollen (*Artemisia* and *Ambrosia*), cat and dog fur, *Alternaria alternata*, cockroaches (*Blatella germanica*), and feather mix. Allergen extracts from Stallergens, France were used.

### 2.3. Statistical Analysis

The statistical analysis was performed using MedCalc Statistical Software version 19.0.3 (MedCalc Software bvba, Ostend, Belgium; https://www.medcalc.org; 2019). Data were labeled as nominal, expressed as percentage, and continuous variables. The normal distribution for continuous variable was done using the Kolmogorov-Smirnov test. Variables with normal distribution were expressed as mean and standard deviation, while variables with abnormal distribution as median and 25–75 percentiles.

The adequate statistic tests according to data distribution were chosen. The differences were assessed within groups by Wilcoxon Signed Rank test and between groups by Mann Whitney test. The χ^2^ test was also used for data analysis. Level of statistical significance was set at *p* < 0.05.

## 3. Results

First of all, the authors calculated the percentage of patients suffering of allergic rhinitis induced by ragweed pollen. The percentage of patients with allergic rhinitis to ragweed was significantly higher in the North-West of Romania compared to the Central region (*p* < 0.001) (see Table 1).

Demographic data of patients with allergic rhinitis to ragweed pollen are presented in Table 2.

Allergic rhinitis to ragweed pollen was most frequently observed in females and in patients living in urban areas, without significant differences between centers. The duration of disease was found to be significantly longer in patients from the Central region compared to patients from the NW region (see Table 2).

### 3.1. Sensitization to Ragweed Pollen

Most of the patients presented polysensitization to different allergens. In the NW region, 27% of the patients (20 pts) were sensitized only to ragweed, while in the Central region, 20.7% (6 pts) presented a positive skin prick test only to ragweed pollen, without statistical differences between groups (*p* = 0.514). Among patients with different sensitization, almost half of them presented co-sensitization to artemisia pollen (48.64% (36 pts) in the NW region and 48.27% (14 pts) in the Central region, *p* = 0.97), or grass pollen (44.59% (33 pts) in the NW region and 44.82% (13 pts) in the Central region, *p* = 0.98). Monosensitized patients were considered if they had sensitization only to ragweed pollen.

There is no correlation between environment (rural-urban), age, sex, family, or personal allergic history and the type of sensitization in patients with allergic rhinitis to ragweed pollen.

### 3.2. Severity of Allergic Rhinitis

**Figure 1:**
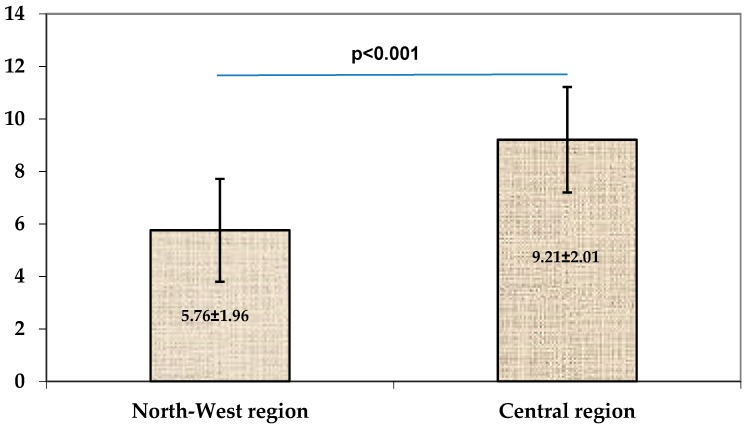
Total symptom scores in both centers (data are expressed as mean ± standard deviation (SD)).

In the NW region, more than half of the patients had mild forms of allergic rhinitis, while in the Central investigational centers, most of the patients presented moderate or severe forms of allergic rhinitis (Table 2). The total symptom score was significantly higher in patients evaluated in the Central region compared to patients from the NW region (Figure 1.)

When the severity of allergic rhinitis was analyzed according to type of sensitization, the authors noticed that patients with monosensitization to ragweed pollen presented more severe forms of disease compared to patients with sensitizations to different pollens in the NW region (*p* = 0.02), but not in the second investigational region (*p* = 0.354) (Table 3). The total symptom score was higher in monosensitized patients in both centers, but the difference reached the statistical significance only in the group from the NW region (*p* < 0.001), not in the Central region group of patients (*p* = 0.16).

There is no association between environment (rural-urban), age, sex, family, or personal allergic history and severity of allergic rhinitis.

### 3.3. Association of Other Allergic Manifestations

The authors also analyzed the association of other allergic manifestations: allergic conjunctivitis and bronchial asthma.

Allergic conjunctivitis was noticed in 41.9% of patients in the NW region, but it was more frequently reported by patients from the Central part, where it was noticed in 75.86% of patients (*p* = 0.02). The allergic conjunctivitis was significantly associated to allergic rhinitis in patients with monosensitization compared to patients with polysensitization in the NW region (*p* = 0.01), but not in the Central region (*p* = 0.12) (Table 4).

Twenty-seven (36.5%) patients from the NW region presented bronchial asthma symptoms, while only 27.6% of patients in the second group (8 pts) presented associated bronchial asthma, although the difference did not reach the level of statistical significance (*p* = 0.39). Patients with multiple sensitization presented more frequently asthma symptoms in the NW group compared to monosensitized patients (*p* = 0.02), but not in the Central group (*p* = 0.51) (Table 4).

## 4. Discussion

The present study characterized the clinical manifestations of allergic rhinitis to ragweed in two distinct regions of Romania, the NW and Central regions. Ragweed has now become an important source of allergens that induces allergic rhinitis in Romania, with an extensive spreading from the West to the Center.

In the present study the sensitization to ragweed pollen was noticed in almost 20% of patients in the NW region, an increased percentage compared to the Central region, knowing that ragweed was not frequently observed in our country until 2007. Ianovici et al. [14] reported a rate of sensitization of 34% in Timisoara in 2009, but this was noticed in the general population, not in patients with clinical manifestations like allergic rhinitis. Recently Florincescu et al. [12] published the first data regarding sensitization to ragweed in patients with allergic rhinitis from Southern part of Romania, reporting a higher percentage of sensitization (48.8%) compared to our study. The highest levels of airborne ragweed pollen in Europe are known to have been recorded in Hungary and Ukraine [6,15], which may explain the higher rate of sensitization from the NW region compared to the Central region. High levels of ragweed pollen have also been recorded in the Black Sea region of Turkey [15,16], which may explain a higher rate of sensitization to ragweed in the Southern region of Romania reported by Florincescu et al. [12]. Looking to all this available data regarding sensitization to ragweed in Romania, we may assume that allergic rhinitis to ragweed could be more frequently observed in Western and Southern parts of the country. Southern and Western parts of Romania are closed to geographical regions rich in ragweed, like Hungary, Ukraine, Bulgaria, and Turkey. The weather conditions (winds, rainfall, average temperatures) in these regions may explain differences in ragweed pollen distribution and secondarily in ragweed sensitization. Ragweed grows intensely on sandy acid soil, which is characteristic for the Southern area of Romania [12,16]. It is an extremely adaptative plant with maximum of germination and multiplication in regions with average temperature around 30 °C in summers [16,17], like in Western and Southern regions of Romania. In summer the wind blows from the West, from Hungary, which may explain a rapid distribution of ragweed pollen from there. In the Central part of the country the average temperature is lower and at the end of summer and autumn the humidity increases, which would explain a reduced growth of the plant and reduced exposure to ragweed pollen [17,18]. Ukraine and Hungary have made extensive attempts to limit ragweed infestation, but Romania only adopted prophylactic measures to limit ragweed spread in 2018.

The distribution of allergy to ragweed could change over the year in all regions of Romania. It would be interesting to analyze the same data over an interval of 5 years to determine the rate of extension of this sensitization, knowing that Florincescu et al. [12] reported an increased number of cases over an interval of 2 years. Actually, an increased rate of sensitization to ragweed was also reported in Germany, the Netherlands, and Croatia [2,19], so a similar increase should be expected in all regions of Romania.

The rate of co-sensitization to other pollens is different in the present research compared to other studies. The co-sensitization to *Artemisia vulgaris* was noticed in almost half of the patients with allergic rhinitis to ragweed, a higher rate than in the Asero study (38%) [20], but significantly lower than the co-sensitization rate observed by Ackermann-Liebrich (82%) [21] in Switzerland. In the present study the co-sensitization to *Artemisia vulgaris* pollen is similar in both centers, an expected result knowing that *Artemisia vulgaris* is a more frequently observed plant in our country compared to the *Ambrosia artemisiifolia*. These results could explain the rapid evolution of sensitization in accordance with the type of exposure to different allergens, old or new, while also considering environmental pollution.

The median age of patients with allergic rhinitis to ragweed was higher in the present study compared to the GAALEN (Global Allergy and Asthma European Network) study [6], but the gender distribution was quite similar that of previously reported data, with this type of allergy being more frequently observed in females compared to males [6,22,23]. An increased median age in the present study is explained by the fact that most of the patients are adults. Children with allergic rhinitis to pollen visit either a pediatric or allergologist specialist, so we may assume that children with allergic rhinitis to ragweed were not included in the present analysis.

In Ackermann-Liebrich study [21], we noticed an increased age of patients with monosensitization compared to those that were polysensitized, but in our study we did not analyze the obtained data on subgroup of patients, due to the small sample size of the groups. Most of the patients lived in urban areas, similar to for previously published data in Europe [3,12,24]. In Florincescu study [12], the rate of patients living in urban area was even higher than in the present study, but in another study [23] the distribution rate was almost equal. It is well known that in industrialized countries, the allergic rhinitis to pollen is more frequently observed in urban areas, due to extensive pollution [1,3].

In the central part of Romania most of the patients presented moderate-severe forms of allergic rhinitis, similar to in the Florincescu study [12], where almost all the patients presented such kind of forms. But surprisingly in NW Romania, most of the patients presented mild forms of rhinitis. Moderate severe forms of allergic rhinitis were reported especially in monosensitized patients rather than in polysensitized patients in the NW, an observation reflected also by a higher total symptoms score. A similar tendency was also noticed in the central region. Persistent moderate and severe forms of allergic rhinitis were also mentioned in previously published data [25,26]. But in a Gelardi study [27] which included only children and not adults, there was no difference compared to the present study regarding the distribution of mild or moderate severe forms of allergic rhinitis to ragweed pollen and mono or polysensitization. We might assume, based on this observation, that ragweed pollen is an aggressive one, especially in adults and produces severe manifestations, which may explain the significant impairment of patient quality of life. Patients with polysensitization may report severe symptoms during other seasons, which is not always related to ragweed pollination.

The association of allergic conjunctivitis to rhinitis symptoms in patients with allergic rhinitis, irrespective of the type of sensitization, is well known [3,22]. The ocular manifestation was more frequently noticed in patients with monosensitization to ragweed pollen in the NW, and was also more frequently reported in Central region. Florincescu et al. [12] reported a similar association rate of conjunctivitis as in the present study in central Romania, higher than we noticed in the NW center. In the association of ocular symptoms, the type and amount of allergen are not the only important factors. Other environmental factors (airborne particles) may also increase the aggressiveness of pollen grains and the ocular response [26,28]. In a Majkowska-Wojciechowska study [28], allergic conjunctivitis is frequently associated in patients from urban areas, but not in those from rural ones. The study center from the central region is an urban area with an increased population, increased traffic, and is more industrialized compared to NW center, which leads to increased pollution.

Bronchial asthma was frequently associated to allergic rhinitis to ragweed, especially in the NW region and in patients with co-sensitizations. A similar rate of association was also reported in previously published data [12,22,24,29]. Surprisingly, Mark et al. [30] reported a lower rate of associated asthma in Hungary, but they did not distinguish between mono- or poly-sensitized patients. Patients with multiple sensitizations are exposed to allergens for longer, which may explain a persistent inflammation in both upper and lower airways and a higher rate of asthma association.

The main strength of this paper is that it presents the first study that characterized the patients with allergic rhinitis to ragweed pollen in two different regions of Romania, with different levels of exposure to allergens. There are also a few limitations of this study. The number of included patients with allergic rhinitis to ragweed is small, especially in the second group from the Central region. Secondly, the authors could not perform a pollen count, so it was not possible to correlate the clinical manifestations of allergic rhinitis or asthma symptoms with pollen level in the air. Also, while the authors used patient files from two centers of Romania, a larger evaluation is needed to determine the prevalence of sensitization.

## 5. Conclusions

Allergic rhinitis to ragweed is a common problem in the NW region of Romania, near Hungary and Ukraine. Ragweed pollen is intensely allergogenic and determines severe forms of allergic rhinitis and association of ocular symptoms. Co-sensitization increases the risk of asthma association. This study recommends including the ragweed pollen in the national panel of the skin prick test to identify the sensitization and to evaluate the clinical manifestation of this allergy. Since the disease is so harmful for human health, it is necessary to limit its geographical extension and to inhibit the growth of the plant in order to protect the general population.

## Figures and Tables

**Table 1:** Patients with allergic rhinitis to ragweed pollen in investigated allergological centers.

Patients	NW Center	Central Center	*p*
Total no. of patients with AR	405 pts	706 pts	
Patient with AR to ragweed	74 pts (18.27%)	29 pts (4.10%)	< 0.001

Abbreviations: AR, allergic rhinitis; NW, North-West; pts, patients.

**Table 2:** Demographic data.

Parameter		NW Center (*n* = 74 pts)	Central Center (*n* = 29 pts)	*p*
Age (age) *		39.5 (31–47)	42.5 (32.5–54)	0.774
Gender ^	M	47.3% (*n* = 35)	44.82% (*n* = 13)	
	F	52.7% (*n* = 39)	55.17% (*n* = 16)	0.530
Living area ^	Urban	77.02% (*n* = 57)	72.41% (*n* = 21)	
	Rural	22.97% (*n* = 17)	27.58% (*n* = 8)	0.808
Severity of AR ^	Mild Moderate/severe	58.10% (*n* = 43) 41.90% (*n* = 31)	10.3% (*n* = 3) 89.7% (*n* = 26)	<0.001
Disease’s duration * (years)		4.25 (2–6)	12 (2–24)	0.009
Family history of allergy		14.90% (11)	17.25% (5)	0.344

* Data are expressed as (median; 25–75th percentile); ^ Data are expressed as (%, *n*); Abbreviations: AR, allergic rhinitis; F, female; M, male; NW, North-West; pts, patients.

**Table 3:** Severity of allergic rhinitis according to the type of sensitization.

Parameter	NW Center			Central Center		
	Monosens. (*n* = 20 pts)	Polysens. (*n* = 54 pts)	*p*	Monosens. (*n* = 6 pts)	Polysens. (*n* = 23 pts)	*p*
AR severity						
Mild Moderate- severe	30% (*n* = 6) 70% (*n* = 14)	68.5% (*n* = 37) 31.5% (*n* = 17)	0.02	0% 100% (*n* = 6)	13.8% (*n* = 4) 86.2% (*n* = 19)	0.354
TSS	7.05 ± 1.83	5.27 ± 1.09	<0.001	10.33 ± 2.07	8.91 ± 2.19	0.16

Abbreviations: AR, allergic rhinitis; monosens, monosensitization; NW, North-West; polysens, polysensitization; pts, patients; TSS, total symptoms score.

**Table 4:** Association of other allergic manifestations.

Parameter	NW Center			Central Center		
	Monosens (*n* = 20 pts)	Polysens (*n* = 54 pts)	*p*	Monosens (*n* = 6 pts)	Polysens (*n* = 23 pts)	*p*
Allergic conjunctivitis	65% (*n* = 13)	33.33% (*n* = 18)	0.01	100% (*n* = 6)	69.56% (*n* = 16)	0.12
Bronchial asthma	15% (*n* = 3)	44.44% (*n* = 24)	0.02	16.7% (*n* = 1)	30.43% (*n* = 7)	0.51

Abbreviations: monosens, monosensitization; NW, North-West; polysens, polysensitization; pts, patients.
